# Hydrologic variability governs GHG emissions in rice-based cropping systems of Eastern India

**DOI:** 10.1016/j.agwat.2024.108931

**Published:** 2024-08-01

**Authors:** L. Arenas-Calle, S. Sherpa, D. Rossiter, H. Nayak, A. Urfels, K. Kritee, S. Poonia, D.K. Singh, A. Choudhary, R. Dubey, V. Kumar, A.K. Nayak, A. McDonald

**Affiliations:** a School of Integrative Plant Science, Soil and Crop Sciences, Cornell University, Ithaca, NY, USA; b CIMMYT-India, Sabajpura, Khagaul, Patna, Bihar 801105, India; c International Rice Research Institute (IRRI), Los Baños, Philippines; d Water Resources Management Group, Wageningen University and Research, Wageningen, the Netherlands; e Environmental Defense Fund, New Delhi 110001, India; f ICAR Research Complex for Eastern Region, Patna, Bihar, India; g ICAR-National Rice Research Institute Cuttack, Odisha 753006, India

**Keywords:** Methane, Mitigation, Landscape hydrology, Dynamic simulation, Indo-Gangetic Plains

## Abstract

Reducing methane (CH_4_) emissions is increasingly recognized as an urgent greenhouse gas mitigation priority for avoiding ecosystem ‘tipping points’ that will accelerate global warming. Agricultural systems, namely ruminant livestock and rice cultivation are dominant sources of CH_4_ emissions. Efforts to reduce methane from rice typically focus on water management strategies that implicitly assume that irrigated rice systems are consistently flooded and that farmers exert a high level of control over the field water balance. In India most rice is cultivated during the monsoon season and hydrologic variability is common, particularly in the Eastern Gangetic Plains (EGP) where high but variable rainfall, shallow groundwater, and subtle differences in topography interact to create complex mosaics of field water conditions. Here, we characterize the hydrologic variability of monsoon season rice fields (*n* = 207) in the Indian EGP (‘Eastern India’) across two contrasting climate years (2021, 2022) and use the **D**e**n**itrification **D**e**c**omposition (DNDC) model to estimate GHG emissions for the observed hydrologic conditions. Five distinct clusters of field hydrology patterns were evident in each year, but cluster characteristics were not stable across years. In 2021, average GHG emissions (8.14 mt CO_2_-eq ha^−1^) were twice as high as in 2022 (3.81 mt CO_2_-eq ha^−1^). Importantly, intra-annual variability between fields was also high, underlining the need to characterize representative emission distributions across the landscape and across seasons to appropriately target GHG mitigation strategies and generate accurate baseline values. Simulation results were also analyzed to identify main drivers of emissions, with readily identified factors such as flooding period and hydrologic interactions with crop residues and nitrogen management practices emerging as important. These insights provide a foundation for understanding landscape variability in GHG emissions from rice in Eastern India and suggest priorities for mitigation that honor the hydrologic complexity of the region.

## Highlights

• Diverse hydrological contexts lead to high variability on GHG emission estimations.
• Blanket mitigation strategies may not be effective for all farmers in Eastern Indian.
• Mitigation potential of irrigation practices is dependent of floodplains topology.
• Interaction of nutrient management with field hydrology drives GHG emissions.

## 1 Introduction

Agriculture, Forestry, and other Land Uses (AFOLU) accounts for 22 % of all anthropogenic greenhouse gas (GHG) emissions, with even higher proportional contributions to methane (47 %) and nitrous oxide (58 %) (IPCC, 2022). Rice cultivation plays a central role in the global methane budget, with roughly 10 % of the global warming potential (GWP) from AFOLU emanating from CH_4_ emissions from rice fields (Wang et al., 2023). Global rice production is concentrated in South and Southeast Asia and these regions contribute an estimated 86 % of the total methane emissions from rice (FAOSTAT, 2023). India is the world’s second-largest rice producer, with annual cultivation spanning 46 million hectares and associated methane emissions estimated at 4.75 Tg CH_4_ per year, constituting approximately 27 % of the global total from rice (FAOSTAT, 2023). Within India, agri-food systems contribute more than 30 % of GHG emissions from all economic sectors (Crippa et al., 2021), with rice ranking second only to ruminant livestock as a GHG source from agricultural production systems (Rao et al., 2019). Furthermore, there is increasing recognition of the importance of mitigating short-lived but potent GHGs like methane that accelerate near-term warming and, hence, the progression to ecological ‘tipping points’ such as forest die-back and melting of permafrost (MacKay et al., 2021, Ocko et al., 2021).

Around 50 % of India's cereals come from the Indo-Gangetic Plain (Shah et al., 2019), with rice-based systems occupying more than 75 % of cultivated area in the Eastern Gangetic Plain (EGP) region, which encompasses parts of India, Nepal, and Bangladesh (Gathala et al., 2020). Gupta et al. (2009) suggests that three out of the four Indian states responsible for the majority of CH_4_ emissions are in the EGP, namely Bihar, Uttar Pradesh, and West Bengal. With high rates of rural poverty, food insecurity, and a largely rural population of nearly 450 million, the EGP (also referred to as ‘Eastern India’ for the purposes of this study) is a priority region for agricultural development (Dutta et al., 2020, Hoque et al., 2023). Rice is primarily grown during the wet monsoon season, which spans from June to October, while crops like wheat and maize are cultivated during the dry winter months from November to April. In addition to constituting the most widely-consumed domestic food staple, India also produces 30 % of the internationally traded rice (FAO, 2022).

Reducing emissions from rice-based systems may provide a powerful pathway for India to make progress towards voluntary GHG mitigation targets (*see* Singh et al., 2022). Nevertheless, there is significative uncertainty about the nature and magnitude of GHG emissions from cropping systems (Ito et al., 2023), particularly from rice given the diversity of production environments ranging from deep water to upland systems (Qian et al., 2023). As a pragmatic approach in data limited environments, most countries conduct national GHG inventories for rice with simplified ‘Tier 1’ equations that estimate methane and nitrous oxide emissions as simple functions of flooding regime and nitrogen use rate (IPCC, 2019). Several studies recognize that these methods are insufficient to capture the spatial variability of GHG emissions at sub-national levels (Peter et al., 2016, Kritee et al., 2018). In India, efforts have been made to move beyond IPCC’s Tier 1 equations by more clearly recognizing the role of different water management practices to emissions, but the resulting categories (e.g., ‘fully flooded’) are only loosely defined and the spatial extent of each category is qualitatively estimated through expert judgement (Bhatia et al., 2013; Gupta et al., 2009; Vetter et al., 2017).

To accurately characterize the nature of GHG emissions from rice systems in Eastern India and appropriately target mitigation solutions, new estimation approaches are required that account for the hydrologic variability of rice fields. Differences in shallow groundwater, topography, and surface drainage networks shape sub-regional to local hydrologic conditions in the region (Bhattacharjee et al., 2022, Singh and Sinha, 2020), and these factors interact with water management and soil parameters to create complex mosaics of field water conditions that vary in space and time, often over short distances (Rossiter et al., 2024). Moreover, every production year is distinct with increasing inter-annual variability in monsoon rainfall over the past two decades (Bhatla et al., 2015, Sharma et al., 2022).

The indivisible connection between field hydrology and GHG emissions in rice is well-established (Souza et al., 2021). Prolonged flooding gradually reduces soil oxygen levels and redox potential, thereby encouraging CH_4_ production from anaerobic respiration. On the other hand, alternating cycles of wetting and drying can induce high rates of N_2_O emissions (Bateman and Baggs, 2005, Butterbach-Bahl et al., 2013, Johnson-Beebout et al., 2009, Kritee et al., 2018). When hydrological variability interacts with soil organic matter and inputs like mineral fertilizers and crop residues, complex patterns of emissions can emerge. The smallholder rice systems of Eastern India are characterized by a high degree management diversity (Kaur et al., 2021, McDonald et al., 2024 *in review*), which further compounds the challenge.

The intersection of hydrologic, soil, and agronomic heterogeneity poses significant challenges to GHG estimation, including for gauging the mitigation benefits associated with agronomic practices such as Alternate Wetting and Drying (AWD) or crop residue recycling in different production contexts. On one hand, *in situ* measurements are expensive and therefore challenging to broadly deploy. On the other hand, empirical models (e.g., carbon calculators based on Tier 1 equations) are relatively easy to deploy but often overlook important drivers of emissions.

To investigate the roles of hydrologic and agronomic management diversity on GHG emissions from rice in the Eastern India, this study has three main objectives. First, we characterize variations in rice hydrology during the monsoon season through field measurements. Then, we simulate GHG emissions with a process-based model of carbon and nitrogen biogeochemistry in agricultural systems (i.e., DNDC – **D**e**N**itrification-**D**e**C**omposition) using observed hydrological data to make inferences about spatial and temporal patterns of emissions. Lastly, we identify the relationships between GHG emissions, field hydrology, and agronomic management towards the goal of creating more robust emission equations for the East India region. With these steps, we endeavor to demonstrate the importance of recognizing the complexity of rice systems in Eastern India to adequately capture the nature of GHG emissions and to prioritize mitigation measures accordingly.

## 2 Material and methods

### 2.1 Study area

This study was conducted in Bihar State and four adjacent districts in Uttar Pradesh State in the EGP region. The Ganges, flowing from west to east, divides Bihar into southern and northern alluvial plains that are traversed by a dozen major tributaries. In this basin, underlying hydrological conditions depend on the interaction of seasonal rainfall patterns with the highly diverse geomorphological and sediment characteristics shaped by deposition from the world’s second largest river (Singh and Pandey, 2014). Annual rainfall in the study region varies between 990 and 1700 mm from year-to-year with more than 80 % of rainfall occurring during the monsoon season from mid-June until early October (Tesfaye et al., 2017). The annual rainfall in the study area exhibited significant differences, rain gauges located in West Champaran recorded with a mean precipitation of 1512 mm in 2021, and 894 mm in 2022 across Bihar, representing a 40 % reduction in rainfall from one year to the next. Rice is typically planted in July and harvested from late October through early November and is directly followed by winter-season crops like wheat or maize that are cultivated during the comparatively dry and cooler winter period.

### 2.2 Data collection

**Fig. 1:**
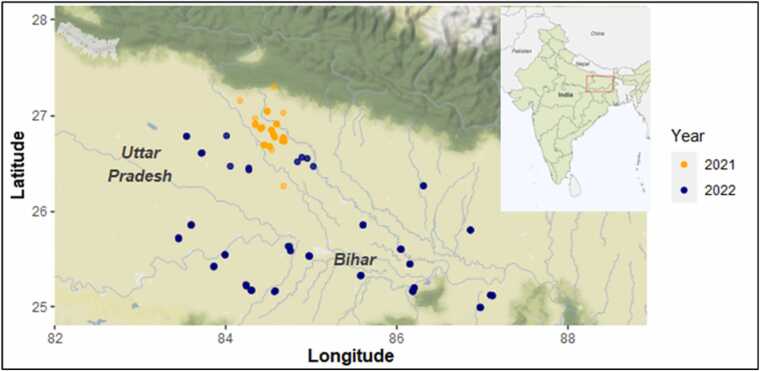
Location of rice production fields in Eastern India where hydrology was monitored through daily measurements. Orange points indicate locations in 2021 with blue points locations across a broader geographic region in 2022.

Field water monitoring tubes were installed across 16 districts in Bihar and 4 districts in Uttar Pradesh in 2022 (*n* = 160 fields), with observations from 2021 (*n* = 47 fields) concentrated in a single Bihar district (West Champaran). Observations were recorded on a daily time-step and reflected either the height of the floodwater above the soil surface or the depth at which saturated soil conditions were present down to 15 cm. Measurements were taken from July 7th to November 11th in 2021, and from July 6th to November 2nd in 2022. For the 2022 sampling campaign, 4–14 tubes were installed per district and were purposively chosen to represent a range of farmer-perceived local differences in hydrological behavior among fields where rice is typically cultivated (Ajay et al.,2022). Sampling locations for both years are depicted in Fig. 1.

The monitoring tubes were constructed using 10 cm diameter plastic PVC pipe with a length of 30 cm. Small (5 mm) perforations were cut into the bottom half of the pipe to permit water infiltration, and the tubes were inserted to a soil depth of 15 cm with the center of the tube kept clear of soil. Water depth was measured every morning from the bottom of the inserted pipe using a measuring tape. The soil surface was designated as zero (0), and measures below the soil surface were recorded as negative values. Positive value indicates depth of floodwater above the soil surface.

Soil, agronomic, and yield data were collected for every field. Soil properties included particle size fractions (clay, silt, and sand), pH, organic carbon (OC; %), and bulk density (BD; g/cm³). Soil methods and protocols used in this study are available online in Sherpa et al. (2022). Management information included sowing and harvesting dates, fertilizer application rates, tillage, and irrigation practices. The agronomic information used in this study is available online and was collected as part of the Landscape Crop Assessment Survey (‘LCAS’) (*see* https://data.cimmyt.org/dataverse/csisadvn; https://systems-agronomy.github.io/lcas/). The LCAS is a collaboration between the Cereal Systems Initiative for South Asia (CSISA) and the Indian Council of Agricultural Research (ICAR) that has collected agronomic data at scale in India since 2017 (McDonald et al., 2024 *in review*).

### 2.3 Simulating greenhouse gas (GHG) emissions

We used the DNDC.v9.5 model to characterize the influence of observed field hydrology conditions (see 2.2) on GHG emissions (CH_4_, N_2_O) from rice systems for two contrasting growing seasons (2021, 2022). DNDC is a process-based model designed for simulating nitrogen and carbon cycles in agro-ecosystems (Li et al., 1992). Simulations were conducted for 47 sites in West Champaran (Bihar state) in 2021 and for 160 sites distributed across 19 districts in Bihar (15 districts) and Utter Pradesh (4 districts) in 2022. We calibrated crop parameters for DNDC according to Partridge et al., (2011) with a stepwise process, beginning with the adjustment of maximum biomass under non-stress conditions, followed by the tuning of thermal degree days (TDD) to match maturity and harvest dates with observed data. The maximum biomass values were derived from multi-year yield records spanning from 2019 to 2021. Grain, leaf, stem, and root fractions (0.46, 0.20, 0.20, 0.15, respectively) were taken from Singh (2013) and Kar et al. (2021), while the optimum rice growing temperature (29°C) was taken from Dhanushka (2011). The crop component was evaluated using a subset of 27 sites from West Champaran in the 2021 season, resulting in a root mean square error (RMSE) for yield of 0.6 t/ha, representing a RMSE of 16 % (*see* Supplementary Information I).

Measured soil properties were used to implement a tailored parameterization of DNDC for each production field, including bulk density (BD), pH, clay fraction, and organic carbon. Additional soil parameters including bulk density (BD), water filled pore space at field capacity and wilting point (WFPS%), and hydraulic conductivity were calculated using pedotransfer functions (PTFs). In cases where BD values were missing for the 2021 sites, they were computed using the PTF developed by Abdelbaki (2018). The estimated BD values were then evaluated against available BD data, resulting in a RMSE of 0.099 g/cm³. Saturated hydraulic conductivity was determined using the PTF proposed by Mohawesh. (2014) and evaluated by Abdelbaki (2021), while the water-filled pore space (WFPS%) at field capacity and wilting point were calculated with the PTFs suggested by Arruda et al. (1987). Porosity was estimated using the standard equation based on bulk and apparent density (2.65 g/cm³). Pedotransfer functions and the porosity equation can be found in Supplementary Information I.

Soil factors were treated as intrinsic attributes and characterized for individual fields, whereas agronomic practices were standardized across sites and represent the average planting dates, harvesting dates, tillage practices, and fertilizer practices recorded in the LCAS data for rice in this region. Nitrogen fertilization included one basal dose of 25 kg N ha^−1^ (applied as DAP) at planting, and two applications of 50 kg N ha^−1^ (applied as urea) top-dressed at 25 and 55 days after sowing. Two tillage passes (plowing at depths of 10 and 20 cm) were implemented one day before planting. Observed daily field hydrology data were used as model inputs for each site, with a water table file generated based on records of water levels in the monitoring tubes during the assessed period. To complete the annual cycle, wheat was simulated during the winter cultivation period, leaving around six weeks of fallow between wheat harvest and rice establishment.

The climate data required to run the DNDC model was obtained from the NASA’s POWER Project (https://power.larc.nasa.gov). These climate datasets encompass daily minimum and maximum temperatures (°C), rainfall (cm), humidity (%), and wind speed (m/s). Based on field locations, a total of six climate files were created for the year 2021, while 23 files were generated for 2022. For rice season simulations, the observed water tube data was used as the hydrologic input instead of precipitation.

To standardize the greenhouse gases according to their global warming potential (GWP), we calculated emissions of each gas in CO_2_-equivalent terms (AR6; IPCC, 2021). This involved multiplying the fluxes of CH_4_ and N_2_O by 27 and 273, respectively, to estimate GWP over a 100-year timescale. The total seasonal GHG flux for each field was then estimated by summing the daily fluxes from sowing to the harvest for each GHG.

It was not possible evaluate GHG simulations against measured field data from Bihar. However, several studies have verified the performance of DNDC in Indian rice systems. Babu et al. (2005) and (2006) evaluated DNDC across 10 sites in India and reported an average relative deviation of 20 % between observed and simulated methane emissions. For a single experimental site with multiple agronomic treatments, Pathak et al. (2005) reported deviations of less than 5 % for methane emissions and 6.8 % for nitrous oxide.

### 2.4 Characterization of hydrological responses

We employed a cluster analysis approach to characterize the major types of hydrologic behavior observed in the 2021 and 2022 rice seasons. Clusters were created based on a set of descriptors that reflect the hydrological characteristics of the water level time series.

#### 2.4.1 Hydrological descriptors

The duration and frequency of floods and drainage periods governs redox reactions and GHG-associated microbiological activity in rice fields (Le Mer and Roger, 2001). To characterize the field water regime in a given field, 17 hydrologic descriptors were calculated from the water tube data and organized into three categories: A) flooding periods; B) dry periods; and C) saturated/unsaturated transitions. A description of these indicators is presented in Table 1.

**Table 1:** Descriptor variables for field water dynamics, grouped into three categories: flood periods, drainage periods, and transitions between saturated and unsaturated conditions.

**Categories**	**Descriptor**	**Units**
**A. Flood periods**	**1.** Percentage of the total period flooded (Above soil level 0 cm)	(%) calculated based on total days of monitoring
	Percentage of days flooded at height of: **2.** 0–5 cm, **3**. 5–10 cm, **4.** >10 cm	(%) calculated based on total days flooded
	Duration of flooding: **5.** one day **6**. within a week **7.** within a month **8.** more than a month	Days; (%) calculated over the total of flood events
**B. Drainage periods**	Number of dry days at depths: **1.** 0 to −5 cm **2.** −5 to −10 cm **3.** <-10 cm	(%) calculated based on total days of drainage
	Average duration (in days) of dry events at depths: **4.** 0 to −5 cm **5.** −5 to −10 cm **6.** <-10 cm	Number of days
**C. Speed of Transition between saturated/unsaturated conditions**	**1.** Number of flood events	Number of days
	**2.** Number of days water level drops from 5 cm to −5 cm in less than 24 hours	Number of days
	**3.** Number of days of quick drainage (water level drop more than 10 cm in less than 24 hours)	Number of days

#### 2.4.2 Cluster analysis of observed hydrological indicators

To identify major hydrological types, we conducted cluster analysis utilizing the K-means algorithm (McQueen, 1967) for each season. This analysis is based on the 17 indicators outlined in Table 1 that were computed for individual fields. The clustering analysis was executed using the R Statistical Software (v4.1.2; R Core Team, 2023) with the ‘Cluster’ package (v2.1.4; Maechler et al., 2023). To determine the optimal number of clusters, we employed the Silhouette index (Rousseeuw, 1987), calculated using the ‘NbClust’ R package (v3.0.1; Charrad et al., 2014). High silhouette coefficient values indicate how well an observation belongs to a particular cluster in comparison to others. In both years, we segregated five clusters, striking a balance between achieving a reasonable number of members in each cluster and ensuring a clear separation of hydrological patterns based on silhouette values. ‘FeatureImpCluster’ (v0.1.5; Pfaffel, 2021) R package was used to identify the most significant descriptors for each cluster. To assess the statistical significance of methane emission differences between hydrologic clusters, we conducted an Analysis of Variance (ANOVA) along with Fisher’s Least Significant Difference (LSD) using the ‘agricolae’ R package (v1.3–6; de Mendiburu, 2023). Furthermore, we performed a correlation analysis between individual hydrological descriptors and GHG estimations for individual fields to determine specific relationships between these descriptors and emissions.

### 2.5 Estimating the influence of field hydrology on methane emissions

To characterize the principal hydrologic drivers of methane emissions, we built a classification tree to predict DNDC-simulated emissions as a function of the aforementioned hydrologic descriptors. The analysis was limited to CH_4_ since methane was the dominant source of GWP in our simulations; also, methane emissions are less sensitive to the idiosyncratic nature of agronomic management differences across farms and fields that can strongly influence N_2_O fluxes such as the timing of nitrogen fertilization, number of application splits, and the field water conditions when fertilizer is applied. Hence, the insights derived for the associations between methane and field hydrology are likely to be comparatively robust across diverse production contexts.

Our methodology combined simulation data from 2021 and 2022 and discretized them into five levels from lower to higher CH_4_ fluxes using the ‘arules’ R package (version 1.7–6; Hahsler et al., 2023). This discretization procedure resulted in the following five emission levels: 1) 0–56.6 kg CH_4_ ha^−1^; 2) >56.6–129 kg CH_4_ ha^−1^; 3) >129–207 kg CH_4_ ha^−1^, 4) >207–317 kg CH_4_ ha^−1^; and 5) >317 kg CH_4_ ha^−1^. We partitioned the dataset into training and evaluation datasets with an 80:20 ratio, ensuring an equitable distribution of each GHG level in both subsets. We first developed a random forest classification model with the ‘RandomForest’ R package (version 4.7–1.1; Liaw, 2002). This analysis was used to assess the relative feature importance and aggregate utility of using hydrological information to predict methane emission in our systems. We also developed a single classification tree using the ‘rpart’ R package (version 4.1.19; Therneau et al., 2022) to visualize interactions among predictor variables.

It is important to note that this study lacks local verification data for GHG emissions. Nevertheless, the primary objective not to create a definitive prediction model, but rather to assess the importance of observed hydrologic variability to relative emission differences between fields and production years. In doing so, we seek to establish the groundwork for ‘simple yet robust’ methods for gauging GHG emissions in rice production environments like those in the EGP where hydrologic complexity is the rule not the exception. And, just as importantly, draw attention to the implications of hydrological variability to both baseline GHG emissions and the roles of different mitigation measures in different production contexts.

### 2.6 Interactions between hydrology, agronomic management, and GHG emissions

We also evaluated the interplay between nitrogen and carbon (i.e., crop residue) management within the context of hydrological variability by performing a sensitivity analysis on representative sites (i.e., the ‘centroid’) for each hydrologic cluster from each year. For the sensitivity analysis, we initiated simulations by increasing the nitrogen use from a baseline rate of 90 kg N ha^−1^ at intervals of 25 % more than the baseline up to 100 % (i.e., 180 kg N ha^−1^); All the split doses increased proportionally, without change the ratio between them. Similarly, for carbon management, the baseline simulations assumed no crop residues were left in the field, and the sensitivity analysis progressively increased the percentage of crop residue retained at intervals of 25 % up to full residue retention.

## 3 Results

### 3.1 Simulating GHG emissions in hydrologically complex landscapes

DNDC simulations revealed significant temporal and spatial variability of GHG emissions. Methane (CH_4_) fluxes were identified as the dominant contributor to the overall GHG emissions (CO_2_-eq), but with significant differences between years for both CH_4_ and nitrous oxide (N_2_O) emissions. Methane estimations were higher in 2021 (wet year) compared to 2022 (dry year), while nitrous oxide emission estimations were more pronounced in 2022 than in 2021. Furthermore, there was spatial variation of total estimated emissions across the surveyed fields within each season, even for the more limited geographical area covered in 2021. The GWP estimates for individual production fields ranged between 0.89 and 16.55 mt CO_2_-eq ha^−1^ in 2021 and, 0.3–10.26 mt CO_2_-eq ha^−1^ in 2022.

**Fig. 2:**
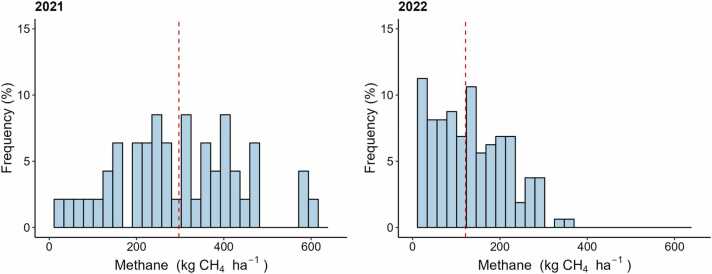
Histogram of cumulative methane (CH_4_) emissions simulated with the DNDC model for 2021 (*n* = 47 fields) and 2022 (*n* = 160). The red dashed lines indicate the mean cumulative flux for each season.

In 2021, average seasonal methane emissions (297 kg CH_4_ ha^−1^) were twice as high as in 2022 (121 kg CH_4_ ha^−1^), with peak emissions reaching 608 kg CH_4_ ha^−1^ for individual fields in contrast to 348 kg CH_4_ ha^−1^ in 2022 (Fig. 2). Seasonal CH_4_ emissions from both seasons ranged from 7 to 608 kg CH_4_ ha^−1^. These values align with previously reported GHG emission measurements from rice-based systems in India which ranged from 4 and 286 kg CH_4_ ha^−1^ across six growing seasons in South India (Kritee et al., 2018) and from 4.85 to 431 kg CH_4_ ha^−1^ across 32 other published experiments as synthesized by Gupta et al. (2021). Additional studies from India reported a mean seasonal CH_4_ emission of 135±23 kg CH_4_ ha^−1^ (Oo et al., 2018). Similarly, Datta et al. (2013) and Datta and Adhya (2014) reported CH_4_ emissions of 282 and 218 kg CH_4_ ha^−1^, respectively.

**Fig. 3:**
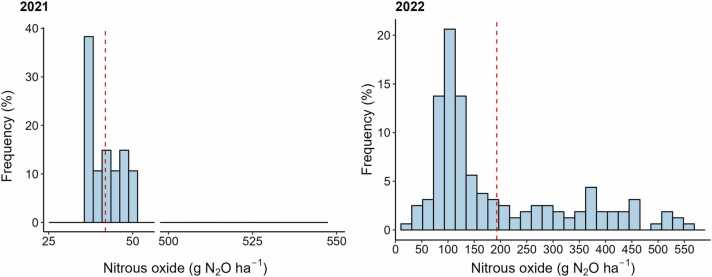
Histogram of cumulative nitrous oxide (N_2_O) emissions simulated with the DNDC model for 2021 (*n* = 47 fields) and 2022 (*n* = 160). The red dashed lines indicate the mean cumulative flux for each season.

During the 2021 season, the average N_2_O estimations were 41.9 g N_2_O ha^−1^, falling within a range of 36.2–49.3 g N_2_O ha^−1^. These values contrasted with 2022, where the average was 192.9 g N_2_O ha^−1^, four times higher than 2021. While this average is still modest compared to the global warming potential of simulated methane emissions, maximum N_2_O emissions in 2022 did reach as high as 550 g N_2_O ha^−1^ (Fig. 3). It is worth noting that nitrogen fertilizer management was standardized for all simulations – same rate, same timing of application. This was done so that the intrinsic differences between sites (i.e., soil and water characteristics) could be more accurately compared without the potential confounding effects of variations in farmer-implemented fertilizer management. In sites with predominantly flooded conditions, model simulated N_2_O emissions just a few days following fertilization, otherwise model will simulate zero N_2_O emissions under flooding conditions.

Model-based N_2_O estimations from 2021 fall within the ranges of previous studies conducted in the Indo-Gangetic Plains. For instance, Ghosh et al. (2003) measured N_2_O emissions ranging from 37 to 187 g N_2_O ha^−1^, while Majumdar et al. (2000) reported values ranging from 34 to 59 g N_2_O ha^−1^. On the other hand, the higher N_2_O model estimations for 2022 do not exceed the maximum values from global (Linquist et al., 2012) and South Asian regional studies where the measured ranges were higher (Kritee et al., 2018; Gupta et al., 2021).

N_2_O emissions in 2021 were estimated to be consistently low across all fields, which corresponds with the generally wet soil conditions in the first two months of the growing season when N fertilizers were applied. In contrast, our simulations for 2022 suggest considerable variability in N_2_O fluxes which corresponds to the observed hydrological differences between fields during the early stages of the growing season and the strong sensitivity of these fluxes to changes from oxidized to reduced soil conditions (Wang et al., 2021).

In aggregate, CH_4_ composed approximately 97 % of all CO_2_-eq emissions across both seasons. Nevertheless, for the driest fields in 2022 (e.g., hydrologic clusters 1–3 – *see* Fig. 5), the relative importance of N_2_O emissions increased to 30 % of total emissions and for some fields constituted the dominant source of global warming potential.

### 3.2 Characterizing field hydrology

To identify typical field hydrology patterns, we performed a cluster analysis for each year based on the flooding and drying indicators described in Table 1. This analysis explained 86 % of the variance in the hydrologic data for 2021 (pseudo-R^2^ = 0.86) and 67 % in 2022 (pseudo-R^2^ = 0.67), with five clusters emerging for each year. We then used feature importance analysis to interpret the role of each hydrologic descriptor for cluster partitioning (see Table 1). We found descriptors associated with flood duration appeared as most important (A5 to A8 descriptors). Descriptors associated with the height of the water table (A2 to A4) play a secondary role. Other descriptors, such as the percentage of the total period flooded (A1), and percentage of total period drained below 10 cm (B3) had a lesser but consistent influence on cluster membership in both seasons.

For drivers of these field hydrology patterns, please refer to (Rossiter et al., 2024) which highlights how multiple factors affect field hydrology. These drivers include soil type, drainage class (i.e., reflecting lateral surface and subsurface flows), groundwater depth, and variations in irrigation practices and precipitation.

#### 3.2.1 Field hydrology in 2021

**Fig. 4:**
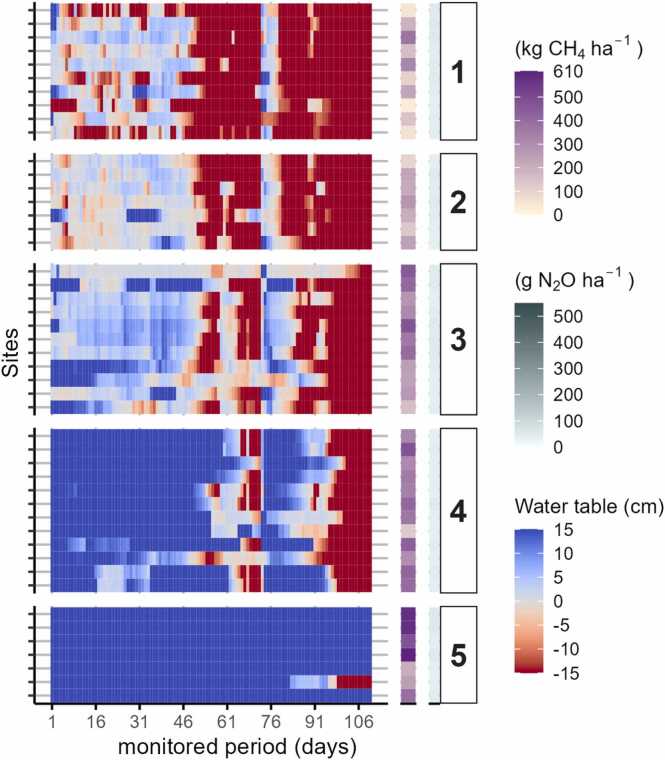
Heatmap of observed daily field water depths (x-axis) along with simulated cumulative CH_4_ and N_2_O emission estimations for 2021. Rows in y-axis represent individual fields that are grouped by hydrologic cluster (#1 - #5, ordered from driest to wettest cluster). Blue tones indicate the presence of floodwater above the soil surface, whereas red tones indicate floodwater recession below the soil surface, with the darkest red tone indicating unsaturated conditions in the top 15 cm of the soil.

In 2021, all the monitored sites exhibited a clear seasonal pattern with some degree of precipitation-driven flooding from late July to the end of September and few fields requiring irrigation (Fig. 4). Notably, 55 % of the sites experienced floods that lasted for more than a month, with some sites were flooded continuously for up to three months. In contrast, floods with a duration of one day represented only 5 % of the total flooded period in 2021. Differences between fields arose primarily from variations in the duration of floods. Cluster #1 represents the driest field conditions observed in 2021, where flooding conditions averaged 27 % of the monitoring period. Cluster #2(’21) and #3(’21) shared similar seasonal patterns and maintained flooding for approximately half of the season with flooding depths typically between 0 and 5 cm. Aside from one sustain drainage event for Cluster #4(’21), Cluster #4(’21) and #5(’21) were consistently flooded with deeper ponded water (>10 cm) for the duration of the season.

#### 3.2.2 Field hydrology in 2022

**Fig. 5:**
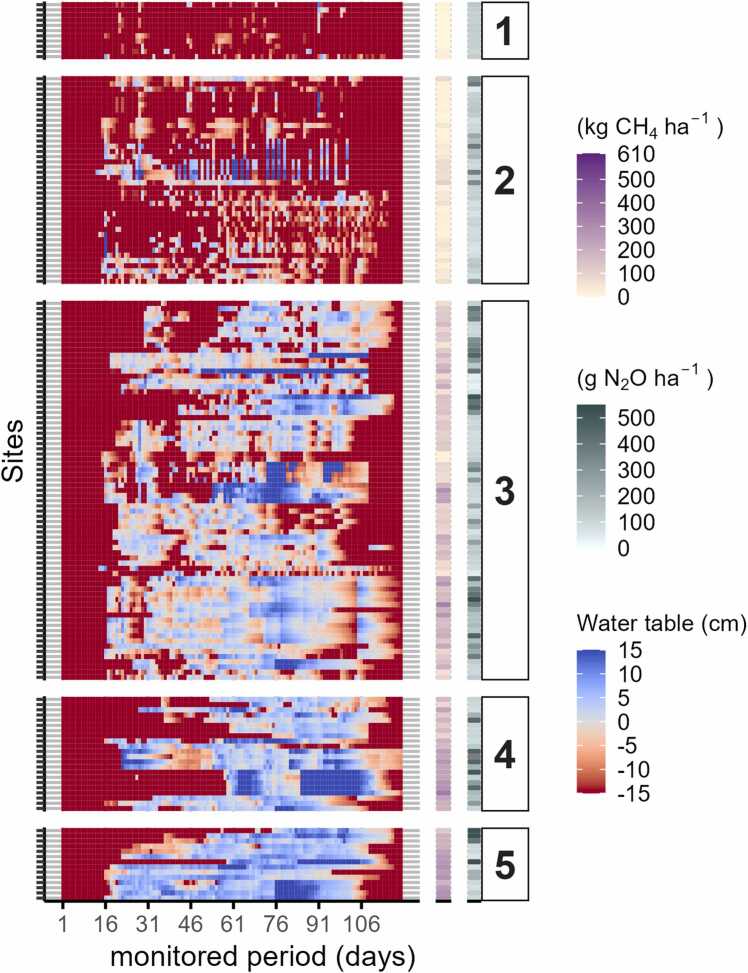
Heatmap of observed daily field water depths (x-axis) along with simulated cumulative CH_4_ and N_2_O emission estimations for 2022. Rows in y-axis represent individual fields that are grouped by hydrologic cluster (#1 - #5, ordered from driest to wettest cluster). Blue tones indicate the presence of floodwater above the soil surface, whereas red tones indicate floodwater recession below the soil surface, with the darkest red tone indicating unsaturated conditions in the top 15 cm of the soil.

All fields were substantially drier in 2022 than those monitored in 2021. In response to lower rainfall, farmers applied between 2 and 30 irrigations during the cropping season, creating a more complex pattern of hydrologic conditions than was evident in 2021, with fields, to different extents, experiencing short-duration flooding that lasted up to a week. Floods with a duration of more than one month occurred in just 11 % of the monitored fields in 2022 (Fig. 5).

The total flooding period for the entire growing season in 2022 ranged from 1 % to 55 % for individual fields. Cluster #1(2022) encompassed the driest sites, characterized by virtually no flooding, and represented less than 10 % of the sites monitored. On the other hand, Cluster #2(’22) was characterized by the most shifts between wet and dry conditions. Cluster #3(’22) was similar in terms of flooding intermittency, but floods, when present, persisted longer; this cluster represents 45 % of the monitored sites. The wettest clusters, #4(’22) and #5(’22), remained flooded for 40 % and 55 % of the growing season, respectively, with fields in Cluster #5(’22) characterized by at least one flooding event lasting more than a month.

#### 3.2.3 Characterizing associations between field hydrology and GHG emissions

**Fig. 6:**
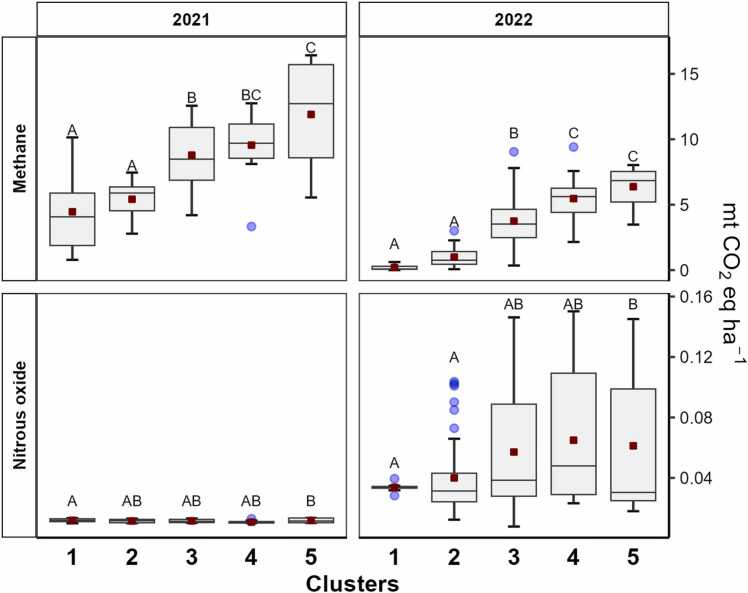
Boxplots of the cumulative emissions for each hydrologic cluster by year (vertical panels) and greenhouse gas (horizontal panels). Clusters are arranged in order from the driest (cluster 1) to wettest (cluster 5) groups of fields which are defined separately for each season. Red symbols indicate average emissions values. Different letters on boxes indicate significant difference (P˂0.05) according to Fisher’s Least Significant Difference (LSD) test.

As depicted in Fig. 4, Fig. 5, simulated GHG emissions varied significantly between hydrology-based clusters (P-value < 0.001). As expected, a positive association between wetter field conditions and increased CH_4_ emissions was predicted across both seasons (Fig. 6). On the other hand, N_2_O emissions were uniformly low within and across clusters in 2021, a result that contrasts with the higher and more varied patterns of N_2_O emissions from the generally drier conditions in 2022. The lack of correspondence between cluster membership and N_2_O emissions in 2021 likely relates to the relatively homogeneous hydrologic conditions observed during the early stages of the rice growth cycle when fertilizers were applied, a scenario that was reversed in 2022 with considerable hydrologic diversity across fields for the same early growth period when reactive nitrogen in the soil system is high and subject to environmental losses.

Based on the significant within-cluster differences in methane fluxes shown in Fig. 6, it is clear that accounting for major differences in season-long hydrologic behavior captures some but not all of the variations in predicted emissions. To gain additional clarity on drivers of emissions, we used Random Forest (RF) and Classification Tree (CT) analysis to predict five different CH_4_ emission levels based on individual descriptors of field hydrology (*see* Table 1), an analysis that combines simulation results from both 2021 and 2022.

The RF model has an OOB (Out Of Bag) classification error of 7.5 %; a confusion matrix is presented in supplementary material_2. Based on Gini Index criteria, the most important predictors are (i) the percentage of the season when the field was flooded (variable A1) and (ii) the percentage of the season when the soil was drained below −10 cm soil depth (variable B3).

**Fig. 7:**
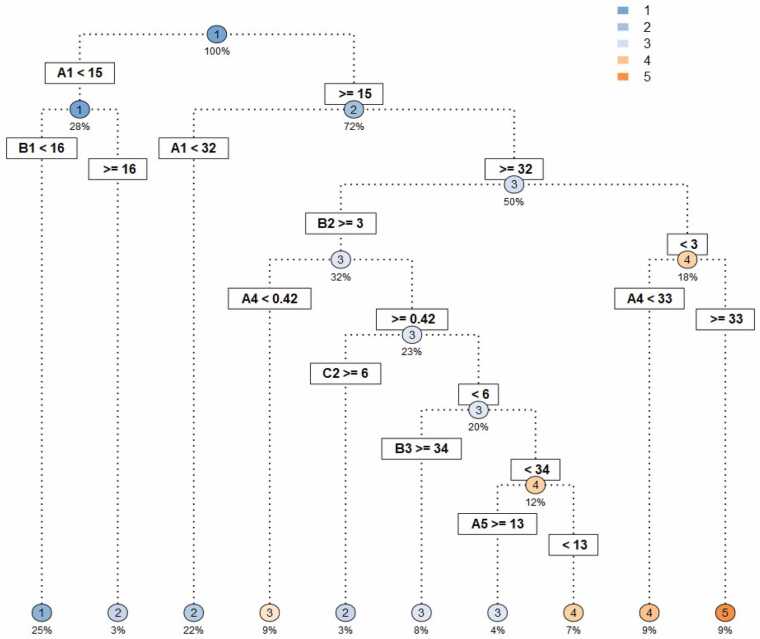
Classification tree for predicted single-season methane emissions: **1)** 0–73.6 kg CH_4_ ha^−1^, **2)** >73.6–162 kg CH_4_ ha^−1^, **3)** >162–254 kg CH_4_ ha^−1^, **4)** >254–383 kg CH_4_ ha^−1^, and **5)** >383 kg CH_4_ ha^−1^. Predictor variables are the hydrologic descriptors presented in Table 1. Variables emerging as important for classifying emissions included: **A1**. Total days flooded (%); **A4.** Percentage of days flooded at height >10 cm; **A5**. Duration of floods of one day duration; **B1**. Days of soil drainage with water between 0 to −5 cm depth; **B2**. Days of soil drainage with water at −5 to −10 cm depth; **B3**. Days of soil drainage with water < −10 cm depth; **B4**. Average duration of drainage periods at 0 to −5 cm; **B5**. Average duration of drainage periods at −5 to −10 cm; and **C2.** Number of days water levels drop from 5 cm above the soil surface to −5 cm below in a single day**.**

To visualize and interpret the relationships between hydrologic indicators and methane emissions embedded in the RF, we then developed a single classification tree (Fig. 7). We found that both the duration and intensity of flooding and draining were crucial for prediction emissions. For example, if total time flooded was less than 15 % of the cropping season and included drainage below 5 cm of the soil (indicator B1), CH_4_ emissions were uniformly low (Level 1). At the other end of the spectrum, the highest (level 5), were predicted when the fraction of season with flooding exceeded 32 % (indicator A1) *and* more than a third of the flood period characterized by deep water conditions (A4). The second split in the classification tree, indicates the importance of seasonal flood duration (A1) for predicting fields in the second lowest emissions level. If this percentage was less than 32 %, emissions are not expected to exceed 162 kg CH_4_ ha^−1^. If more than 32 %, a broad range of emission outcomes are possible depending on the depth and duration of shallow soil drainage (e.g., indicators A4, A5, B2, B3 and C2).

In contrast to the hydrologic descriptors, soil parameters such as clay content, bulk density (BD), and background soil organic carbon (SOC) levels had weak correlations with simulated variations in CH_4_ emissions. However, N_2_O estimations did exhibit a moderate correlation with differences in clay content of individual rice production fields (*data not shown*).

### 3.3 Hydrologic interactions with N fertilizer and crop residue management

For the DNDC simulations presented in Section 3.3, nitrogen fertilizer management was standardized across all simulated fields and crop residues were not returned to the soil, a practice that represents prevailing farmer management in our study region (Ajay et al., 2022). Nevertheless, fertilizer management in Eastern India varies considerably across fields (McDonald et al., 2024 in review) and there are emerging incentives for farmers to return crop residues to the soil to build carbon stocks (Urban et al., 2023).

We ran a second set of simulations to explore the interaction of soil hydrology with nitrogen rate and rice crop residue management on GHG emissions. A representative field from each hydrologic cluster (i.e., the cluster ‘centroid’ – 5 fields from 2021, 5 fields from 2022) was used in the analysis. We focused the analysis on the impact of N fertilization on nitrous oxide emissions and crop residue management on methane emissions.

**Fig. 8:**
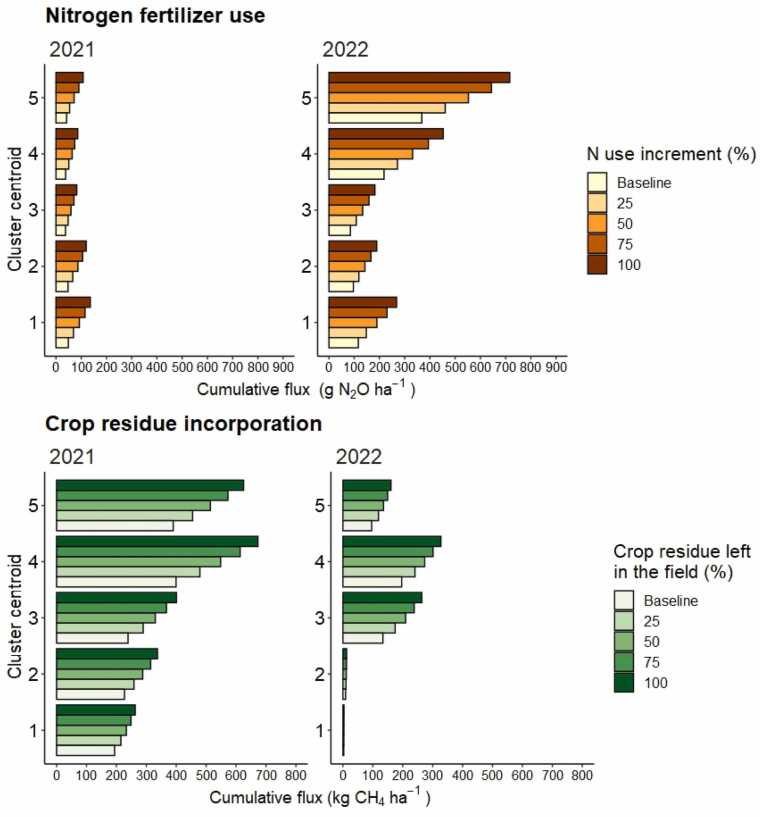
Sensitivity of nitrous oxide emissions to changes in nitrogen fertilizer use (panel A) and methane emissions to percentage of crop residue returned to the soil (panel B) across hydrologic conditions in 2021. Clusters are ordered from driest (cluster #1) to wettest (cluster #5) soil conditions. The baseline nitrogen fertilizer rate is 125 kg ha^−1^ and baseline crop residue return rate is zero.

For N_2_O emissions we found that increased nitrogen rate resulted in larger emissions, especially so in the dryer year of 2022. For the comparatively wet year of 2021, increasing nitrogen fertilizer rates resulted in a small but consistent increase in N_2_O emissions across hydrologic clusters, albeit from a low base (Fig. 8, upper panel). On average, N_2_O emissions increased by 11–22 g N_2_O ha^−1^ for each 25 % increment of additional N, with the most substantial absolute increase observed in the driest fields (i.e., cluster #1). In 2022, the drier of the two years, N_2_O emissions increased between 24 and 85 g N_2_O ha^−1^ for each 25 % increment of additional N from a much higher baseline than in 2021. Somewhat counterintuitively, the largest N_2_O increase in 2022 were simulated for the wettest clusters.

For methane, we found that crop residue only had an impact on emissions in the wetter year with little impact during the drier year of 2022. In 2021, methane emissions in the wetter hydrologic clusters (#3, 4, and 5) increased by an average of 52.8 kg CH_4_ ha^−1^ from a high base when all crop residues were retained. In contrast, the average increase was only 21 kg CH_4_ ha^−1^ in clusters #2 and #1, respectively. In 2022, average methane emissions were much lower and close to zero irrespective of crop residue management in the driest clusters (#1, #2). On the other hand, significant increases in CH_4_ were simulated when all residues were retained in the wetter hydrologic clusters (i.e., average increase of 21.3 kg CH_4_ ha^−1^).

## 4 Discussion

Rice production systems contribute almost half of the global warming potential from cropland on a global basis, primarily from methane emissions (Carlson et al., 2017). China, India, and Indonesia have been identified as the main country-level sources of GHG emissions from rice (Qian et al., 2023). The relationship between soil water and GHG emissions is well-established, with processes like nitrification, denitrification, and methanogenesis all directly linked to the duration and frequency of saturated soil conditions that strongly influence soil oxygen levels and microbially-mediated redox reactions (Marschner, 2021). The central influence of soil water regime on emissions of both CH_4_ and N_2_O emission has also been documented in the context of Indian rice systems (Kritee et al., 2018).

Recent global meta-analyses suggest that methane emissions may decrease by > 50 % with the use of water management practices such as alternate wetting and drying (Jiang et al., 2019). Other analysis suggests that the more generalized concept of ‘non continuous flooding’ (NCF) for water management could be adopted by 76 % of rice farmers on a global scale, with similar transformative reductions in GWP depending on the intensity and timing of drying events (Bo et al., 2022). Underpinning these assessments is the assumption that rice fields are principally maintained in flooded conditions, and that the main challenge to achieving GHG mitigation goals through improved water management is posed by the practical difficulties of draining rice fields (Qian et al., 2023). With this logic, ‘baseline’ GHG emissions for a particular rice production environment are commonly estimated under fully flooded conditions on experiment stations (*see* Bo et al., 2022). National-scale greenhouse gas inventories for India take a more nuanced view of hydrology by segregating three different categories for irrigated systems and associated emission factors. However, these categories are very broad (e.g., irrigated continuously flooded, irrigated single drainage, irrigated multiple drainage events) with estimates of areal extent based primarily on expert judgement (Gupta et al., 2009, Vetter et al., 2017).

The Indo-Gangetic Plains are a dynamic fluvial region with active floodplains, channels, and interfluves (Sinha et al., 2005; Pal et al., 2009). In this study of monsoon-season rice production in the Eastern Ganges Plain, we document tremendous heterogeneity in rice field hydrology that varied spatially and temporally within and across seasons. The hydrologic differences observed in 2021 largely depended on landscape controls on water movement and areas of accumulation, whereas the record dry monsoon season in 2022 compelled farmers to rely more on irrigation, resulting in hydrologic conditions shaped largely by the interaction of variable rainfall and management practices (Rossiter et al., 2024).

Across two contrasting climate seasons, our data suggest that fields with continuous flooding represent a small minority of fields in the EGP region, and heterogeneity appears to be the rule rather than the exception with five different major types of hydrology characterized for each season with cluster analysis. Notably, there was no overlap in hydrologic behavior between the two seasons. This implies a reconsideration of methods used for ‘bottom up’ baseline emission estimates as well as the types of GHG mitigation strategies that fit different production contexts in Eastern India. For example, if most fields already experience non-continuous flooding, changes in water management to increase the frequency of drained conditions would have a less important role for reducing emissions vis-à-vis baseline conditions than is suggested by evidence from research trials (e.g., Bo et al., 2022).

We use the DNDC model constrained by observed field water data to assess the implications of the observed hydrologic diversity in the EGP for baseline GHG emissions and to explore interactions with carbon (i.e., crop residue) and fertilizer management. Why is this approach important? The observed patterns emerge from the complex interaction of topography, soils, rainfall, drainage, irrigation, and shallow groundwater systems (Rossiter et al., 2024); these interactions cannot be reliably simulated with process-based models of the field water balance that only account for one-dimensional water inputs (i.e., rainfall and irrigation) and outputs.

The hydrological patterns characterized in this study are estimated to create a 'patchwork' of GHG emissions across the landscape with notable differences, ranging from 0.006 to 16.55 tons CO_2_-eq ha^−1^, observed even over very short distances (e.g., at the scale of individual villages). Eastern India and the Eastern Gangetic Plains are a priority region for agricultural development for the Government of India (Biswas et al., 2023). As such, understanding and addressing emissions from rice in the EGP as these systems are intensified may ultimately form an important component of India’s nationally determined contribution to greenhouse gas mitigation.

A landscape approach for hydrologic characterization not only provides a more realistic understanding of current emissions but also helps define achievable mitigation goals with changes in agronomic practices. Most studies suggest that changes in water management provide the essential foundation for GHG mitigation strategies in rice (Wassmann et al., 2000; Sanchis et al., 2012; Jiang et al., 2019). Our results suggest a more tempered view, conditioned by the recognition that most fields in the EGP appear to already experience significant periods of drying and, for those that remain fully flooded in a wet year, water is not actively managed by farmers but rather accumulates and stagnates in poorly-drained parts of the landscape.

Beyond water management, there are other near-term pathways to reduce GHGs in rice systems, namely changes to nitrogen and carbon management (Hassan et al., 2022, Qian et al., 2023, Shcherbak et al., 2014, Zhao et al., 2019). However, both pathways have strong interactions with field hydrology (Kritee et al., 2018, Urban et al., 2023). The association between the persistence of soil flooding and methane is an urgent concern since many contemporary mitigation efforts in India focus exclusively on increasing soil organic carbon (SOC) as a sink for atmospheric CO_2_ without considering impacts on methane emissions. Some studies report mitigation benefits from increasing SOC in rice-based systems, while others find that higher CH_4_ emissions overwhelm these gains (Grace et al., 2012, Urban 2024).

To explore the interactions between hydrology and N and C inputs in the EGP, additional DNDC simulations of GHG emissions were conducted with the same observed field hydrology data from 2021 and 2022 but with different scenarios of rice residues management (i.e., carbon) and nitrogen fertilizer rates assessed. As expected, results revealed countervailing GHG emissions responses when higher rates of nitrogen and carbon were added to rice fields with disparate hydrologic conditions. For the driest cluster in each season, N_2_O emissions markedly increased when nitrogen rates increased but had negligible increases in CH_4_ emissions when higher rates of rice residues were returned to the soil. The opposite pattern was simulated for the wettest cluster in both seasons, with much higher CH_4_ emissions and modest increases in N_2_O. These results suggest that knowledge of prevailing hydrologic conditions can form an integral part of GHG mitigation targeting in the EGP with, for example, precision nutrient management and soil carbon accumulation with crop residue recycling prioritized for drier parts of the rice landscape.

Nevertheless, it is important to avoid drawing overly simplistic conclusions about the nature of these general patterns. Our results suggest that hydrology around the time of fertilization rather than season-long water regime mediated ‘hot moments’ for N_2_O emissions, a result consistent with other studies (Akiyama et al., 2005, Jain et al., 2016). In some cases, N_2_O and CH_4_ emissions were relatively high in the same fields in the same season. But these results also indicate that it may be possible to reduce N_2_O emissions by ensuring soil flooding through irrigation for relatively narrow windows around the time of fertilization in the EGP without markedly increasing CH_4_. For methane, our simulation suggests that there is no single answer to the question of net GHG benefits from returning higher rates of crop residues to soils in the EGP; the change in CH_4_ emissions can be nominal (near zero) or profound (> 50 % higher), depending on spatial and seasonal differences in field hydrology.

Results from our DNDC simulations suggest that ‘next generation’ approaches and models for GHG estimation are required that better embrace hydrologic complexity to reliably establish baselines, target management solutions, and to understand the context-dependent mitigation value of these solutions in the rice systems of the EGP. As a first approximation for this approach, we developed a classification tree model for simulated methane emissions based on hydrologic indictors. We initially limit the model to methane since CH_4_ showed a clearer correspondence with hydrological descriptors. Results of this exercise demonstrate the power of indicators such as the period of the growing season when fields are flooded to estimate emissions. By combing ground observations with enhanced process-based simulations, we anticipate that it will be possible to further refine ‘simple yet robust’ emission factors (i.e., new Tier 2 equations) for rice systems in the region, including for the relatively complex challenge of estimating N_2_O emissions which may be underestimated in the current study as compared to the measured emissions ranges reported in Kritee et al., (2018).”. Field research trials and associated GHG measurements have been initiated in the EGP to verify and potentially improve the model-based emission predictions described herein.

To guide GHG mitigation strategies, key attributes of rice production fields (e.g., hydrology, fertilizer management) must be estimated at scale with reasonable accuracy and cost. Recent studies have demonstrated the power of using remotely-sensed hydrology data to estimate methane emissions in relatively homogenous rice production environments such as the Mekong Delta (Arai et al., 2018). The forthcoming NASA-ISRO Synthetic Aperture Radar (NISAR) Mission (see Kellogg et al., 2020), will provide new capabilities to efficiently characterize hydrologic characteristics at fine spatial and temporal scales, an advance that is needed to leverage remote sensing to aid GHG mitigation efforts in the complex rice production system of the Eastern Ganges Plain.

## 5 Conclusions

Rice systems in the Eastern Ganges Plain have been identified as a ‘hot spot’ for greenhouse gas emissions. Our study uses observed field water data to constrain model-based estimates of GHGs, with results suggesting that hydrologic diversity in these systems has a profound impact on net greenhouse gas emissions. This diversity occurs within and across production seasons and emerges from an interacting set of drivers including landscape features and management practices, with only a small minority of fields remaining flooded for the duration of the growing season. Effective climate change mitigation strategies for rice in the EGP region must embrace hydrologic complexity to set intervention priorities and to target context-dependent solutions.

The study compared modeling results with past reports on greenhouse gas (GHG) emissions in the region. However, future simulations could be enhanced by conducting more comprehensive model evaluations. The broad spectrum of GHG estimates, affected by diverse hydrological conditions interacting with the inherent uncertainty of 1-D water balance models, indicates that neglecting landscape heterogeneity might restrict our grasp of model performance. Therefore, the study suggests bolstering GHG monitoring networks in the study area to facilitate modeling-driven studies in the future.

## Declaration of Competing Interest

The authors declare that they have no conflicts of interest.

## Data Availability

Data will be made available on request.
