# Supplementary material for: Hydrologic variability governs GHG emissions in rice-based cropping systems of Eastern India
Source: Agric Water Manag. 2024 Aug 1;301:108931. doi: 10.1016/j.agwat.2024.108931 (PMC11304473; doi:10.1016/j.agwat.2024.108931)
Supplement: Supplementary file 1 — Supplementary material [file mmc1.docx]

***Supplementary material_1***

**Hydrologic variability governs GHG emissions in the rice-based cropping systems of Eastern India**

***Table S1****. Default and calibrated crop parameters in DNDC*

| **Crop parameters** | **Default** | **Calibrated** |
| --- | --- | --- |
| Max yield (kg C/ha) | 5200 | 2920 |
| Biomass fraction  (leaf/stem/root/grain) | (0.24/0.23/0.41/0.12) | (0.22/0.22/0.46/0.10) |
| Biomass C/N ratios  (leaf/stem/root/grain) | (85/85/85/45) | (85/85/85/45) |
| accumulative thermal degree days (TDD; °C) | 2000 | 4100 |
| Optimum Temp (°C) | 25 | 29 |
| Water requirement (g water/g DM) | 508 | 508 |
| N fixation index | 1.05 | 1.05 |


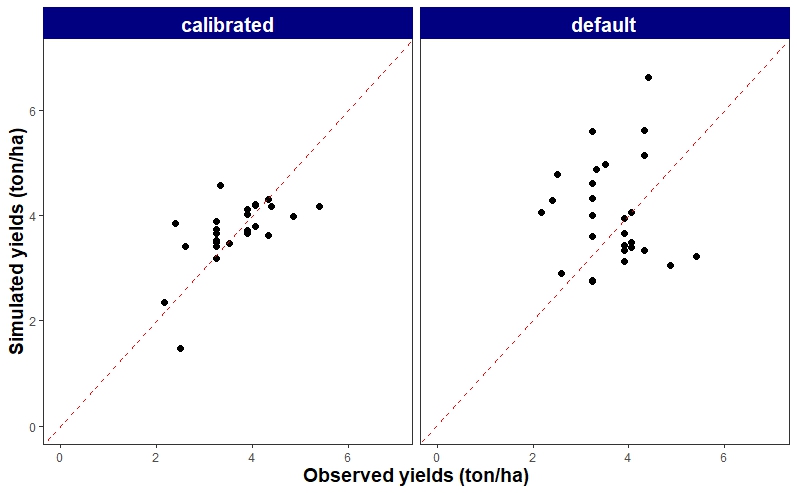


***Figure S1.*** *Comparison of observed yields for 2021 cropping season in West Champaran with simulated yields in DNDC, each panel represents the simulated values sung default crop parameters and the right panel the simulations aftercrop calibration.*

**Pedotransfer functions and standard equations used to estimated soil parameters required in DNDC**

Bulk density; Abdelbaki. (2018):

$Bulk density \left( \frac{g}{{cm}^{3}} \right)=1.449*e^{-0.03*SOC (\frac{g}{kg}soil)}$ *eq.1*

Saturated hydraulic conductivity; Mohawesh. (2014) in Pali et al. (2009):

$K_{sat}=360000*{10}^{((-0.006*sand\left( \% \right))-4.875)}$ *eq.2*

Water-filled pore space (WFPS%) at field capacity and wilting point; Arruda et al. (1987):

$WFPS\left( \% \right)_{Field capacity}=\left( 0.29*\left( \left( clay\left( \% \right)+silt\left( \% \right) \right)+9.93 \right) \right)*Bulk density (\frac{g}{{cm}^{3}})$ *eq.3*

${WFPS\left( \% \right)}_{wilting point}=(0.27*(((clay\left( \% \right)+silt\left( \% \right))+1.07)$) * $Bulk density (\frac{g}{{cm}^{3}})$ *eq.4*

Porosity calculated using a standard equation based on bulk and apparent density (2.65 g/cm³):

$Porosity \left( \% \right)=\left( 1-\frac{Bulk density \left( \frac{g}{{cm}^{3}} \right)}{2.65 \left( \frac{g}{{cm}^{3}} \right)} \right)*100$ *eq.5*

**References**

Abdelbaki, A. M. (2018). Evaluation of pedotransfer functions for predicting soil bulk density for U.S. soils. *Ain Shams Engineering Journal*, *9*(4), 1611–1619. <https://doi.org/10.1016/j.asej.2016.12.002>

Abdelbaki, A. M. (2021). Selecting the most suitable pedotransfer functions for estimating saturated hydraulic conductivity according to the available soil inputs. Ain Shams Engineering Journal, *12*(3), 2603–2615. <https://doi.org/10.1016/j.asej.2021.01.030>

Arruda, F.B., Julio, J. Jr., & Oliveira, J.B. (1987). Parametros de solo para calculo de agua disponivel com base na textura do solo. Revista Brasileira de Ciência do Solo. 11, 11-15.

Mohawesh O.E. (2014). Development of pedotransfer functions for estimating soil retention curves and saturated hydraulic conductivity in Jordan valley. Jordan Journal of Agricultural Sciences. 10:67–82. <https://doi.org/10.12816/0029875>.
