# Supplementary material for: Hydrologic variability governs GHG emissions in rice-based cropping systems of Eastern India
Source: Agric Water Manag. 2024 Aug 1;301:108931. doi: 10.1016/j.agwat.2024.108931 (PMC11304473; doi:10.1016/j.agwat.2024.108931)
Supplement: Supplementary file 2 — Supplementary material [file mmc2.docx]

***Supplementary material_2***

**Hydrologic variability governs GHG emissions in the rice-based cropping systems of Eastern India**

**Feature of importance based on misclassification rate:**

**Figure S2-1**. Feature of importance 2021. A1) Percentage of the total period flooded; A2) Percentage of days flooded at depths of 0-5 cm; A3) Percentage of days flooded at depths of 5 to 10 cm; A4) Percentage of days flooded at > 10 cm; A5) Duration of flooding for less than 1 day; A6) Duration of flooding within a week; A7) Duration of flooding within a month; A8) Duration of flooding for more than one month; B1) number of dry days at depth -5 to 0 cm; B2) number of dry days at depth -10 to -5 cm; B3) number of dry days at depth <-10cm; B4) Average duration (in days) of dry events at depths 0 to -5 cm; B5) Average duration (in days) of dry events at depths -5 to -10 cm; B6) Average duration (in days) of dry events at depths <-10 cm; C1) Number of flood events; C2) Number of days water level drops from 5 cm to -5 cm; C3) Number of days of quick drainage (water level drop more than 15 cm).

**Figure S2-2**. Feature of importance 2022. A1) Percentage of the total period flooded; A2) Percentage of days flooded at depths of 0-5 cm; A3) Percentage of days flooded at depths of 5 to 10 cm; A4) Percentage of days flooded at > 10 cm; A5) Duration of flooding for less than 1 day; A6) Duration of flooding within a week; A7) Duration of flooding within a month; A8) Duration of flooding for more than one month; B1) number of dry days at depth -5 to 0 cm; B2) number of dry days at depth -10 to -5 cm; B3) number of dry days at depth <-10cm; B4) Average duration (in days) of dry events at depths 0 to -5 cm; B5) Average duration (in days) of dry events at depths -5 to -10 cm; B6) Average duration (in days) of dry events at depths <-10 cm; C1) Number of flood events; C2) Number of days water level drops from 5 cm to -5 cm; C3) Number of days of quick drainage (water level drop more than 15 cm).

**Random forest model outcomes:**


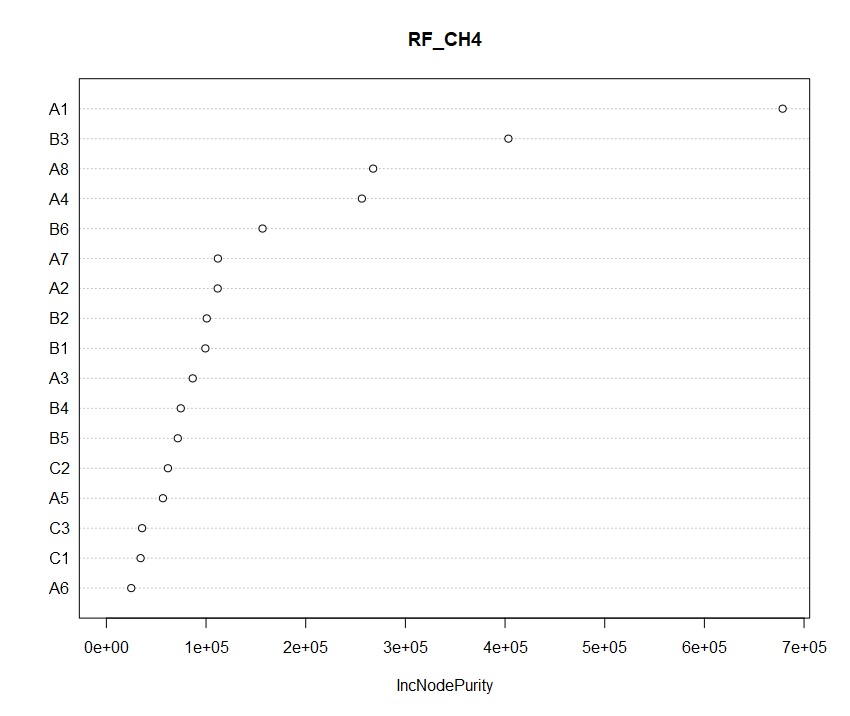


**Figure S2-3**. Variables of importance in Random Forest (RF) model. A1) Percentage of the total period flooded; A2) Percentage of days flooded at depths of 0-5 cm; A3) Percentage of days flooded at depths of 5 to 10 cm; A4) Percentage of days flooded at > 10 cm; A5) Duration of flooding for less than 1 day; A6) Duration of flooding within a week; A7) Duration of flooding within a month; A8) Duration of flooding for more than one month; B1) number of dry days at depth -5 to 0 cm; B2) number of dry days at depth -10 to -5 cm; B3) number of dry days at depth <-10cm; B4) Average duration (in days) of dry events at depths 0 to -5 cm; B5) Average duration (in days) of dry events at depths -5 to -10 cm; B6) Average duration (in days) of dry events at depths <-10 cm; C1) Number of flood events; C2) Number of days water level drops from 5 cm to -5 cm; C3) Number of days of quick drainage (water level drop more than 15 cm).
